# Modulators of epithelial-mesenchymal transitions in prostate cancer: potential for novel therapeutics development

**DOI:** 10.3389/fphar.2026.1808307

**Published:** 2026-08-18

**Authors:** Jinxia Zheng, Ling Li, Holly Russell, Duygu Duzgun, Sebastian Oltean

**Affiliations:** Department of Clinical and Biomedical Sciences, University of Exeter Medical School, Exeter, United Kingdom

**Keywords:** cancer cell migration, E-cadherin, epithelial-mesenchymal transitions, naltrexone hydrochloride, N-cadherin, novel therapeutics, prostate cancer

## Abstract

Research in the area of hallmarks of cancer has opened the possibility of designing novel therapeutic interventions based on modulating cancer properties. Interestingly, the epithelial-mesenchymal transition (EMT), important in both tumour growth and metastasis, has not been targeted in prostate cancer (PCa). Previously, in a repositioning screen, three new chemicals that modulate EMT in PCa (LLSO1 - NNC-55-0396 dihydrochloride; LLSO2 - Nemadipine A; LLSO3 - Naltrexone hydrochloride) have been found. This study aims to investigate how they work mechanistically as well as the effect of these LLSOs on the properties of PCa cells both *in vitro* and *in vivo*. Although these LLSOs exhibited varying effects on different cancer cell lines, all of them modulated EMT by inhibiting the migration of PCa cells. LLSO3 also exhibited a significant inhibitory effect on tumour growth *in vivo*, concomitant with an increase in the expression levels of E-cadherin. Further investigation was conducted to obtain mechanistic insights into the LLSO compounds mode of action. In conclusion, our work strongly supports that LLSO compounds may become important candidates for targeted therapeutics in prostate cancer.

## 1 Introduction

Prostate cancer (PCa) is the most commonly diagnosed cancer in men in the Western world and is the second most common cancer in men globally (Sung et al., 2021; IARC, 2024; Bray et al., 2018). In UK, more than 50,000 men are diagnosed with this disease, and more than 12,000 men die from PCa every year. One in eight men will be diagnosed with prostate cancer in their lifetime, and around 510,000 men are living with or after PCa in UK (Lloyd et al., 2015; Cancer Research UK, 2024). While recent progress has been made in the last years in the treatment and management of PCa, considering the high incidence and high number of deaths, much effort is still required to find new targeted treatments.

More than 20 years ago, Hanahan and Weinberg developed an excellent framework to organize and understand the myriad of cancer properties–the hallmarks of cancer (Hanahan and Weinberg, 2000). This has been very useful in cancer biology in the search for key targets and therapies. Despite its central role in cancer progression, epithelial–mesenchymal transition (EMT) remains a hallmark of cancer for which no effective targeted therapies exist.

EMT is the process through which epithelial cells transition to a mesenchymal phenotype, with the reverse–mesenchymal-to-epithelial transitions–also occurring. EMT is prominently active during embryogenesis, enabling cells to transition from epithelial to mesenchymal states for migration, before re-establishing epithelial characteristics to form new structures. EMT is shut down mainly in adult life with the exception of processes such as wound-healing. In addition, the EMT program regulates some immune cells' properties. In the context of cancer, EMT and the reverse MET are thought to be some of the most important mechanisms for cancer progression and metastasis (Kalluri and Weinberg, 2009). Consequently, intense efforts have been made to develop therapies to target EMT.

Despite the recognized role of EMT in cancer progression, invasion, metastasis, and therapy resistance, EMT remains an under-targeted therapeutic axis in PCa. While EMT has been extensively studied in cancers such as breast, colorectal, and lung cancer, translational efforts to develop EMT-targeted therapies for PCa have been limited for several reasons. First, one major challenge is that EMT is a dynamic and reversible phenotypic program regulated by multiple redundant signalling networks, including TGF-beta, Wnt, Notch, receptor tyrosine kinase, MAPK/ERK, PI3K/AKT, and hypoxia-associated pathways (Periyasamy et al., 2007; Goshima et al., 2019; Wendt et al., 2014; Xu et al., 2015; Arisan et al., 2020), making it difficult to inhibit using single-target approaches. Second, canonical EMT transcription factors such as Snail, ZEB and Twist are difficult to inhibit directly because they are nuclear proteins and often lack conventional druggable enzymatic domains. Third, broad inhibition of central pathways, such as TGF-beta, can lead to systemic toxicity due to the pleiotropic roles of these pathways in both human physiology and cancer development (Kang et al., 2006). These challenges support the need to identify alternative pharmacological strategies that modulate EMT-associated phenotypes without relying on direct inhibition of core EMT transcription factors.

In prostate cancer, EMT regulation is further complicated by androgen receptor signalling, tumour heterogeneity, and lineage plasticity, which allow tumour cells to transition between epithelial and mesenchymal states during disease progression and therapy resistance. Although several EMT-modulating strategies have been proposed, including targeting signalling pathways, transcription factors, or microRNAs, none have been clinically validated in prostate cancer. This represents a significant therapeutic gap, particularly because EMT is associated with increased migration, invasion, metastasis, and resistance to androgen-targeted therapies. Therefore, identification of pharmacological agents capable of modulating EMT through alternative and potentially druggable signalling pathways may provide new opportunities for therapeutic intervention in prostate cancer.

In previous work, we have designed a bi-fluorescent reporter based on alternative splicing of the fibroblast growth factor receptor 2 (FGFR2) and demonstrated that it could be used as a sensor of EMT in cancer cells (Oltean et al., 2008). More recently, using this reporter, we performed a repositioning screen using the LOPAC library to find compounds that can act as modulators of EMT. We identified three compounds (LLSO1 - NNC-55-0396 dihydrochloride; LLSO2 - Nemadipine A; LLSO3 - Naltrexone hydrochloride) and conducted preliminary validations (Li et al., 2022).

LLSO1 (NNC-55–0396 dihydrochloride) is a selective T-type calcium channel inhibitor. T-type calcium channels have been implicated in cancer cell proliferation, survival, angiogenesis and tumour growth (Welsby et al., 2003; Choi et al., 2005; Zhang et al., 2017; Sallán et al., 2018). NNC-55-0396 has been reported to inhibit proliferation and induce apoptosis in T-type calcium channel-expressing leukaemia cell lines, and to inhibit angiogenesis through suppression of hypoxia-inducible factor-1alpha signalling (Huang et al., 2015; Kim et al., 2015). LLSO2 (Nemadipine A) is an L-type calcium channel antagonist originally identified through a small-molecule screen in *Caenorhabditis elegans* (Kwok et al., 2006). Nemadipine A has also been linked to apoptotic sensitisation in cancer cell models, it has demonstrated pro-apoptotic effects in cancer models (Kwok et al., 2006). LLSO3(Naltrexone hydrochloride) is an opioid receptor antagonist, primarily known for blockade of mu-opioid receptor signalling, although opioid receptor biology in cancer is complex and context-dependent (Scroope et al., 2021; Carli et al., 2020; Lennon et al., 2014; Tripolt et al., 2021). It has been reported to inhibit EMT and suppress migration and invasion in several cancer types by blocking opioid receptor–mediated signalling pathways, primarily the mu-opioid receptor (Liu and Dalgleish, 2022; Wang et al., 2021). Collectively, although these compounds have demonstrated anti-proliferative or anti-angiogenic properties, their effects on EMT modulation in PCa have not been systematically examined. Addressing this gap is particularly relevant given the absence of clinically validated EMT-targeted therapies in PCa and the central role of EMT in metastatic progression and therapeutic resistance.

Here, we show that these three compounds modulate EMT-associated markers, focusing on E-cadherin and N-cadherin expression in prostate cancer cells as well as some other cancer cell lines. Furthermore, we describe their mechanism of action and how they influence properties of cancer cells such as migration, growth or apoptosis; we also demonstrate inhibition of tumour growth *in vivo* in PCa xenografts using LLSO3 treatment. We also include preliminary inhibitor-based experiments to explore signalling pathways that may contribute to LLSO1- and LLSO3-associated changes in E-cadherin expression. The study is presented as a phenotypic and preliminary mechanistic characterisation of EMT-modulating compounds, rather than as definitive proof of direct pathway mechanisms.

## 2 Materials and methods

### 2.1 Cell lines and cell culture

PC3 and DU145 prostate cancer cell lines were obtained from Microvascular Research Laboratories (MVRL), University of Bristol. LNCaP prostate cancer cells were purchased from the American Type Culture Collection (ATCC, Manassas, VA, USA). MDA-MB-231 and MCF-7 breast cancer cell lines; CALU-3 non-small-cell lung cancer cells; HCT-116 colorectal carcinoma cells; and SKOV-3 and A2780 ovarian cancer cells were all obtained from the Clinical and Biomedical Sciences Laboratories (CBS), University of Exeter.

PC3, DU145, LNCaP, SKOV3, and A2780 cells were maintained in RPMI 1640 (Sigma) supplemented with 10% Fetal Bovine Serum (FBS, Gibco) and 1% Penicillin streptomycin (Sigma). MCF-7, MDA-MB-231, HCT116, and CALU-3 cells were cultured in Dulbecco’s Modified Eagle’s Medium (DMEM, Sigma-Aldrich) with 10% FBS (Gibco) and 1% Penicillin streptomycin (Sigma). All cell lines were incubated under standard conditions: a humidified incubator at 37 °C with 5% CO_2_. All cell lines were maintained for fewer than approximately 10 passages after thawing and were used during logarithmic growth phase for all experiments.

### 2.2 Western blot

Cells were lysed with a RIPA lysis and extraction buffer (Thermofisher) containing 1% Halt proteinase inhibitor cocktail (Thermofisher). Protein samples were denatured in Laemmli sample buffer (Bio-Rad) with 2-Mercaptoethanol (Bio-Rad), total proteins were loaded onto TGX stain-free protein gels (Bio-Rad), for Western transfer to PVDF membranes (Bio-Rad) and immunoblotting. Following this, membranes were blocked with 5% BSA in 50 mm Tris-HCl (pH 7.4), 150 mm NaCl, and 0.3% Tween 20 for 1 h, immunoblots were probed overnight at 4 °C with the following primary antibodies: anti-E-cadherin (Abcam, ab231303, 1:1000), anti-N-cadherin (Abcam, ab18203, 1:1000), and anti-Twist (GeneTex, GTX127310, 1:500). After washing, membranes were incubated with a fluorescent secondary antibody (Li-Cor) for an hour and imaged using a Licor imager. Blots were analysed by quantitative densitometry using Bio-Rad ImageLab software. Protein expression levels were normalised to total protein signal from the corresponding lane to account for differences in protein loading and transfer efficiency. For each target, the normalised value for each treatment group was then expressed relative to the DMSO-treated control group.

### 2.3 Immunofluorescence

Cells were seeded onto coverslips in 6-well plates and allowed to adhere for 24 h. Following treatment with either DMSO or various LLSOs for 48 h, cells were fixed with ice-cold methanol for 10 min and permeabilized with 0.05% Triton X-100 (Thermofisher) in PBS for 10 min, followed by permeabilization. Nonspecific binding sites were blocked with PBS containing 1% Bovine Serum Albumin (BSA, Sigma-Aldrich) and 5% normal goat serum (Abcam) for 1 h at room temperature. Cells were then incubated with either Alexa Fluro 488 Goat anti-Mouse IgG (Invitrogen) as the isotype control or anti-E-cadherin (BD Biosciences, 612131, 1:1000), and anti-N-cadherin (Abcam, ab18203, 1:1000) primary antibodies in PBS with 1% BSA overnight at 4 °C. After three washes with PBS, cells were incubated with Alexa Fluor 488 goat anti-mouse secondary antibody (Invitrogen) for 90 min and mounted in VECTASHIELD with DAPI (VECTASHIELD® Antifade Mounting Medium with DAPI, Vector Labs). Fluorescence images were acquired using Leica DM4000 LED fluorescence microscopy and image analysis was performed using ImageJ software. For each condition, fluorescence intensity was measured from 9 randomly selected microscopic fields. The mean fluorescence intensity for each treatment group was normalised to the background fluorescence using control regions without cells. Images compared within the same experiment were acquired using identical microscope settings, including magnification, exposure time, and illumination intensity. The number of independent experiments, replicate wells, and analysed fields are stated in the corresponding figure legends.

### 2.4 Migration assay (Boyden chamber assay)

Cells were pre-treated with either DMSO as control, LLSO1 5 µM, LLSO2 10 µM, or LLSO3 10 µM for 48 h. LNCaP cells were subsequently serum-starved overnight in serum-free medium, while DU145 cells were cultured without starvation due to their rapid migration characteristics. After treatment and cell starvation, 150,000 treated cells in serum-free medium were seeded into Millicell hanging cell culture inserts (Merck) placed in 24-well plates containing complete medium with 10% FBS as a chemoattractant. Following 24 h of incubation, non-invasive cells that stayed in the interior of the insert were removed by gently wiping with a moistened cotton swab. Migrated cells on the outer side of the membrane were fixed and stained with DAPI. Cell counts were obtained from three random fields per insert using fluorescence microscopy. Data were analysed by one-way ANOVA with Dunnett’s multiple comparisons test using GraphPad Prism 9.

### 2.5 Apoptosis assay (JC-10 mitochondrial membrane potential assay)

Cells were seeded in 90 μL of media per well in a black-walled, clear-bottom 96-well culture microplate and cultured overnight at 37 °C and 5% CO2. Following treatment with DMSO or various concentrations of LLSOs for 48 h, the JC-10 assay kit (Abcam) was used according to the manufacturer’s protocol. Fluorescence intensity was measured using the SpectraMax M2e Multi-mode Plate Reader with the following settings: Ex/Em = 490/525 nm (cut off at 515 nm) and 540/590 nm (cut off at 570 nm) for ratio analysis.

### 2.6 Population growth curve of the combination treatment

50,000 of PC3 cells or 70,000 of DU145 cells were seeded per well in 24-well plates. After 24 h, cells were treated with DMSO as a control, 5 µM LLSO1, 10 µM LLSO2, 10 µM LLSO3, 5 µM LLSO1 + 10 µM LLSO2, 5 µM LLSO1 + 10 µM LLSO3, 10 µM LLSO2 + 10 µM LLSO3 or 5 µM LLSO1 + 10 µM LLSO2 + 10 µM LLSO3. Cells were counted every 24 h for a total of 120 h, with media and treatments refreshed every 48 h. Data were analysed with GraphPad Prism 9 and tested by two-way ANOVA with Dunnett’s multiple comparisons test.

### 2.7 Mice xenografts

All the *in vivo* experiments were approved by the University of Exeter Animal Welfare and Ethical Review Body (AWERB) and conducted in accordance with UK legislation. CD-1 nude mice (Charles River Laboratories, Wilmington, MA, USA) were subcutaneously injected with 3 million PC3 cells in 100 µL PBS (n = 12). Once tumours reached 3 mm in diameter, mice were randomised into two groups (n = 6/group) and injected intraperitoneally three times weekly with either saline (control) or 10 mg/kg LLSO3. Tumour size was measured three times weekly using calipers, and volume was calculated as [(length + width)/2] × length × width. Once the tumour size reached the allowed endpoint with a diameter of 12 mm in any direction, mice were culled by cervical dislocation and the tumours were extracted. Tumours were photographed before being flash frozen in liquid nitrogen for further analysis. Statistical significance was assessed by using Two-way ANOVA through GraphPad Prism 9.

### 2.8 Quantitative polymerase chain reaction (qPCR)

Total RNA was extracted by TRIzol reagent (Ambion) and total RNA (2ug) from each sample was reverse-transcribed with GoScript Reverse Transcription Mix and random primers (Promega) following the manufacturer’s instructions. Quantitative real-time PCRs (qPCR) was performed using PowerUp SYBR Green Master Mix (Thermofisher) on a LightCycler 96 Real-Time PCR System (Roche). The following oligonucleotides were used: E-cadherin F: 5’-TCC​GAA​GCT​GCT​AGT​CTG​AG-3’; R: 5’-CTC​AAG​GGA​AGG​GAG​CTG​AA-3’. The oligonucleotides used for normalization were GAPDH F: 5’-GTC​AGT​GGT​GGA​CCT​GAC​CT-3’; R: 5’-TGA​CAA​AGT​GGT​CGT​TGA​GG-3’. There are two repetitions within each of the 3 independent tumour samples. n = 6. Results were expressed as Mean ± SD, *p < 0.05, **p < 0.01.

### 2.9 LC50 (MTT)

PC3 cells (10,000/well) were seeded in a 96-well plate and incubated at 37 °C and 5% CO2 for 24 h. After cells were treated with either DMSO or different concentrations of LLSO for 48 h, the MTT reagent (Abcam) was added per the manufacturer’s instructions, including wells without cells as background controls. Absorbance at OD = 590 nm was measured using a SpectraMax M2e Multi-mode Plate Reader, with blank-corrected values used to calculate viability. The data were analyzed through GraphPad Prism 9 by using one-way ANOVA with Dunnett’s multiple comparisons test.

### 2.10 Alamar blue assay

Cells (10,000/well) were seeded in a 96-well plate for 24 h, then treated with either DMSO, 5 μM LLSO1, 10 μM LLSO2 or 10 μM LLSO3. After 48 h of treatment, viability was assessed using AlamarBlue reagent (Thermofisher) per the manufacturer’s instructions, with fluorescence measured on a SpectraMax M2e plate reader (Molecular Devices). Data were analyzed through GraphPad Prism 9 by using one-way ANOVA with Dunnett’s multiple comparisons test.

## 3 Results

### 3.1 LLSOs modulate the expression of EMT markers in prostate cancer cells

**FIGURE 1:**
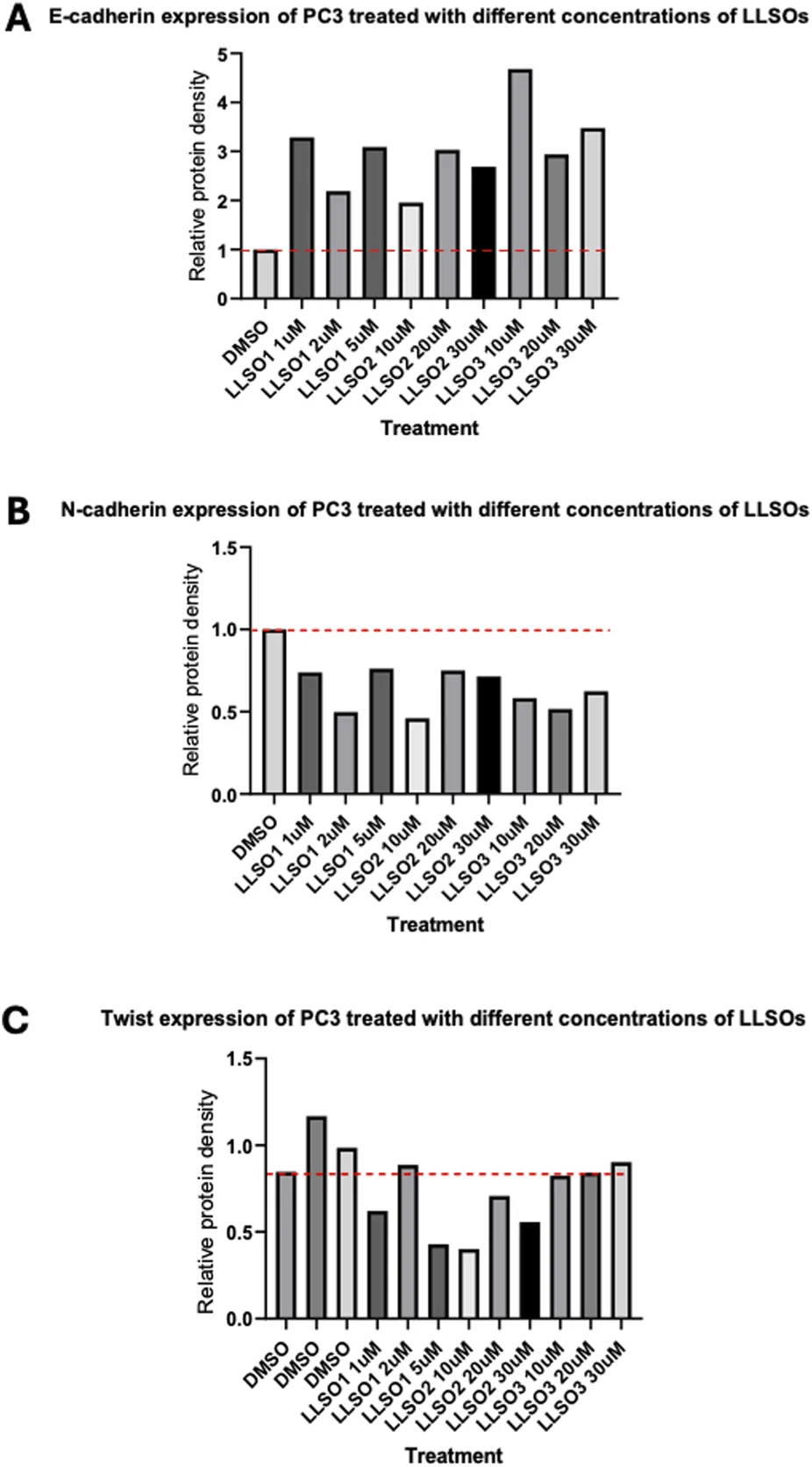
LLSOs increase the expression of E-Cadherin and decrease expression of N-cadherin and Twist in PC3 cells, as assessed by Western blot. **(A)** Quantification of E-Cadherin protein levels in Western blot from PC3 cells treated with DMSO (control) or different concentrations of LLSOs. All three chemicals showed increased the levels of E-cadherin expression. N = 2, The experiment was repeated twice, data was analysed by one-way ANOVA. **(B)** Quantification of N-Cadherin protein levels in Western blot from PC3 cells treated with DMSO (control) or different concentrations of LLSOs. All three chemicals showed decreased the levels of N-cadherin expression. N = 1, The experiment was done once, data was analysed by one-way ANOVA. **(C)** Quantification of Twist protein levels in Western blot from PC3 cells treated with DMSO (control) or different concentrations of LLSOs. All three chemicals showed decreased trend on Twist expression level. N = 1, The experiment was done one time. Data was analysed by one-way ANOVA.

We first assessed whether LLSO1, LLSO2 and LLSO3 altered EMT-associated marker expression in PC3 prostate cancer cells. Concentrations close to the previously determined LC50 (LC-lethal concentration) were selected (Supplementary Figures S1-S3), with the aim of evaluating EMT-associated phenotypes while avoiding interpretation based solely on extensive cytotoxicity. Western blot analysis suggested increased expression of the epithelial marker E-cadherin following treatment with the LLSO compounds. Conversely, the mesenchymal-associated markers N-cadherin and Twist showed reduced or downward-trending expression following treatment (see quantifications in Figure 1 and blots in Supplementary Figures S4-S6).

**FIGURE 2:**
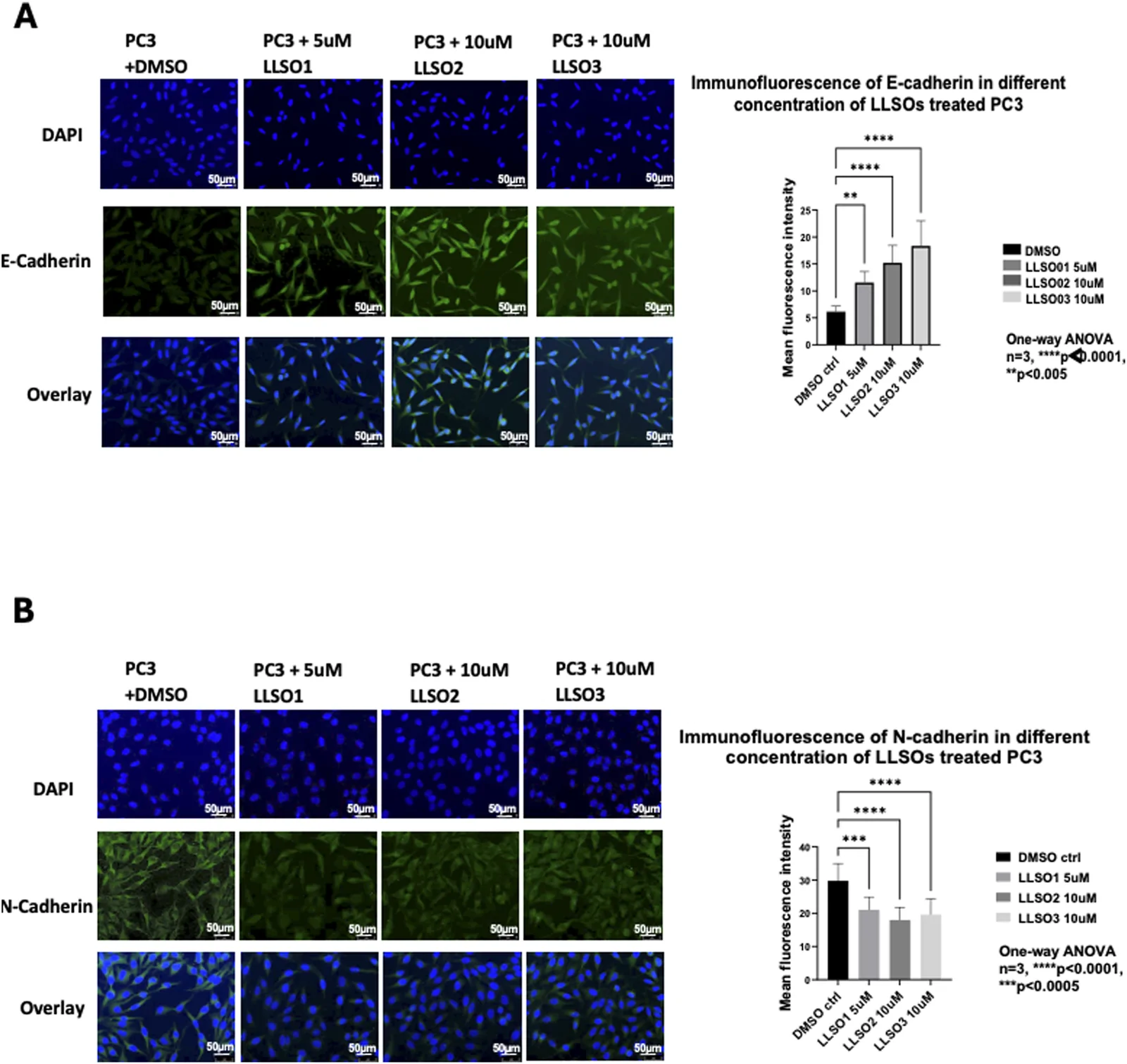
LLSOs increase the expression of E-cadherin in PC3 cells, as assessed by immunofluorescence. Immunofluorescence was performed following 48 h of pre-treatment with DMSO or 5 µM of LLSO1, 10 µM of LLSO2 and 10 µM of LLSO3 in PC3 cells. **(A)** *Left panel* - Representative fields taken at ×200 magnification; *Right panel* - Normalized E-cadherin expression of LLSO1 5µM, LLSO2 10µM, LLSO3 10 µM treated PC3 cells and DMSO (as control) treated PC3 cells. N = 3, Representative examples from at least 9 microscopic fields per experiment, experiments repeated 3 times with triplicate per treatment. Pictures were captured at ×200 magnification, and the scale bar is 50 µm. Data were analysed by one-way ANOVA **(B)** *Left panel* - Representative fields taken at ×200 magnification; *Right panel* - Normalized N-cadherin expression of LLSO1 5µM, LLSO2 10µM, LLSO3 10 µM treated PC3 cells and DMSO (as control) treated PC3 cells. n = 3, Representative examples from at least 9 microscopic fields per experiment, experiments repeated 3 times with triplicate per treatment. Pictures were captured at ×200 magnification, and the scale bar is 50 µm. Data were analysed by one-way ANOVA.

The expression of EMT markers was also assessed by immunofluorescence. Figure 2 shows an increase in E-cadherin fluorescence and decrease in N-cadherin when PC3 cells are treated with LLSOs. Similar results, albeit with smaller changes in expression levels, were obtained in another prostate cancer cell line–DU145 (see Supplementary Figures S7, S8).

We therefore concluded that indeed the LLSO compounds are modulators of EMT at non-toxic concentrations.

### 3.2 The effects of LLSO compounds on prostate cancer cell function

We next investigated how LLSO compounds affect prostate cancer cell behaviours, both EMT-related and independent.

Previous data from our group showed that both LLSO1 and LLSO3 reduced cell growth rate, all three LLSOs inhibited cell migration, and LLSO1 decreased the proliferation rate of PC3 cells. The effects of LLSOs on the properties of LNCaP and DU145 cells *in vitro* were verified.

**FIGURE 3:**
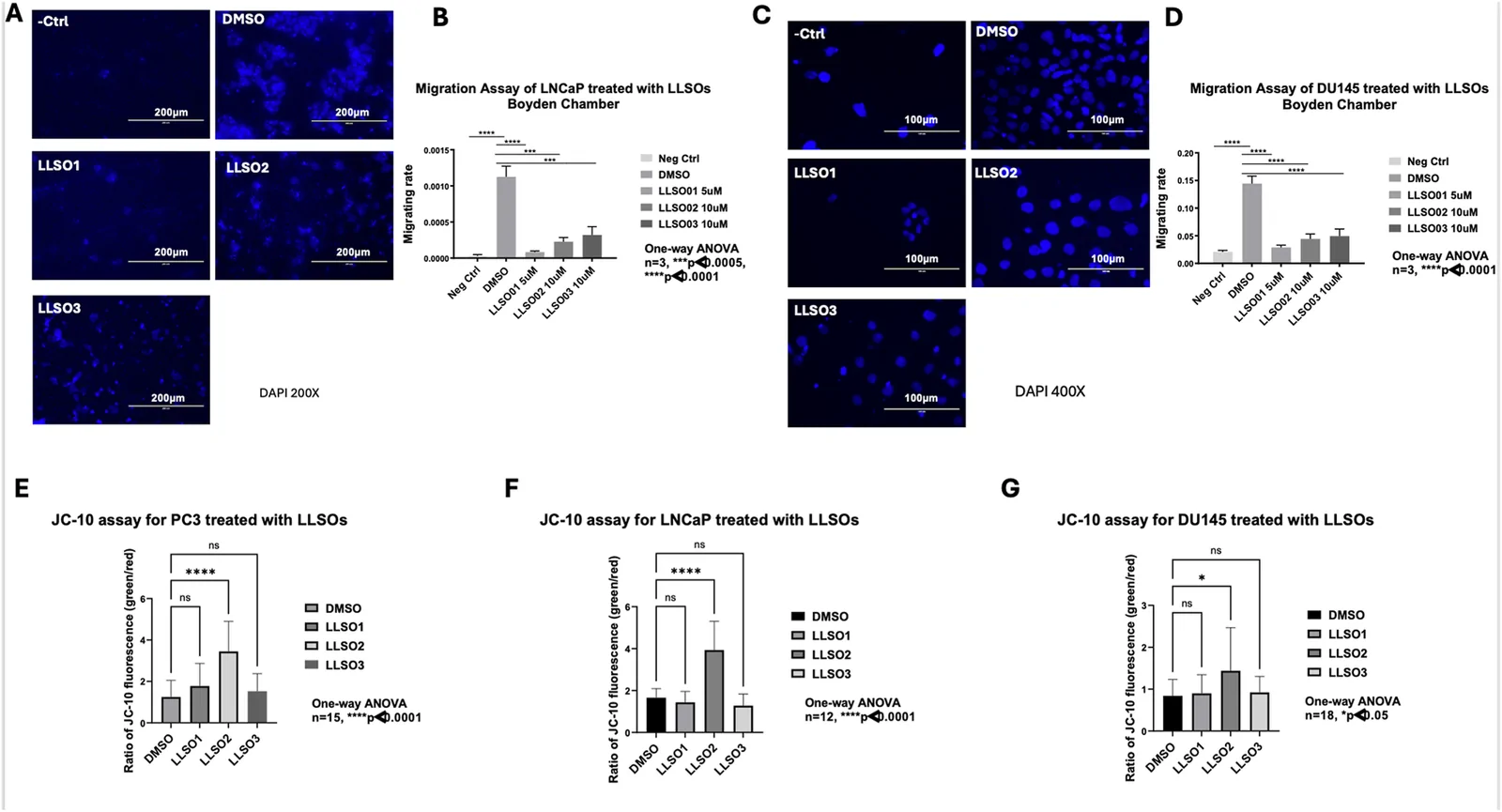
LLSOs decrease the migration rate in both LNCaP and DU145 cells and LLSO2 induces apoptosis in PC3, LNCaP, and DU145 cell lines. Boyden Chamber assay was performed following 48 h of pre-treatment of LNCaP and DU145 cells with DMSO and LLSOs. LNCaP and DU145 cells were cultured for 24 h in the reduced medium for starving cells. Representative images for LNCaP cells were taken at ×200 magnification, and ×400 magnification for DU145 cells. **(A)** Representative photographs of Boyden chamber assay for either DMSO or one of the LLSOs for pre-treated LNCaP cells. LNCaP cells not starved were used as the negative control. **(B)** Normalized migration rate on Boyden chamber assay of 5 µM LLSO1, 10 µM LLSO2, 10 µM LLSO3 or DMSO treated LNCaP cells by counting cells on the Photomicrographs. Data were analysed by one-way ANOVA. experiment was repeated three times with three replication per treatment in each individual experiment, ***p < 0.0005, ****p < 0.0001. **(C)** Representative photographs of Boyden chamber assay for either DMSO or one of the LLSOs for pre-treated DU145 cells. DU145 cells not starved were used as the negative control. **(D)** Normalized migration rate on Boyden chamber assay of LLSO1 5µM, LLSO2 10µM, LLSO3 10 µM and DMSO treated DU145 cells by counting cells on the Photomicrographs. Data were by one-way ANOVA, three individual experiments with three replication per treatment in each individual experiment, ****p < 0.0001. Lower panel: JC-10 assay was performed following 48 h pre-treatment with 5 µM LLSO1, 10 µM LLSO2, 10 µM LLSO3 or DMSO of PC3, LNCaP and DU145 cells, as well as pre-treatment with 5 mM H2O2 as the positive control. Normalized apoptosis ratio on JC-10 assay of either DMSO control or one of the three LLSOs treated **(E)** PC3, **(F)** LNCaP and **(G)** DU145 cells by using the fluorescence intensity ratio of green to red fluorescence. Data were analysed by one-way ANOVA, *p < 0.05, ****p < 0.0001. For PC3, three individual experiments with five replicates per treatment in each individual experiment, n = 15; For LNCaP, two individual experiments with six replicates per treatment in each individual experiment, n = 12; For DU145, three individual experiments with six replicates per treatment in each individual experiment, n = 18.

All three LLSOs have been shown to inhibit cell migration in PC3 cells using the Boyden chamber assay (Li et al., 2022). As shown in Figures 3A–D, all LLSOs significantly decreased the migration rate for both LNCaP and DU145, as expected given their EMT-inhibitory activity.

The potential of LLSOs to induce cell apoptosis in PC3, LNCaP, or DU145 was evaluated using the JC-10 mitochondrial membrane potential assay. As shown in Figures 3E–G, LLSO2 induced cell apoptosis in all cell lines, whereas both LLSO1 and LLSO3 did not affect apoptosis in any of these PCa cell lines. These results suggest that the compounds differ in their effects on mitochondrial membrane potential and cell death-associated readouts.

Growth curve analysis further showed compound- and cell line-specific effects. As shown in Supplementary Figure S9, 5 µM LLSO1 was toxic in LNCaP cells (though this is similar to the LC50 for LLSO1 in PC3 cells). The growth of LNCaP cells was inhibited by LLSO1, and all cells were killed after 120 h of exposure to 5 µM LLSO1. Both 10 µM LLSO2 and 10 µM LLSO3 showed a significant reduction in LNCaP growth. Similarly, 5 µM LLSO1(Supplementary Figure S10) also decreases significantly in the growth of DU145 cells while there is no significant effect for both 10 µM LLSO2 and 10 µM LLSO3.

The Alamar Blue assay was performed to assess the effect of LLSOs on cell viability in PC3, LNCaP, or DU145 cells. As shown in Supplementary Figure S11, the three LLSO treatments had no significant effects in both PC3 and DU145 cells, while they slightly reduced cell viability in LNCaP cells.

### 3.3 LLSOs have cumulative effects

**FIGURE 4:**
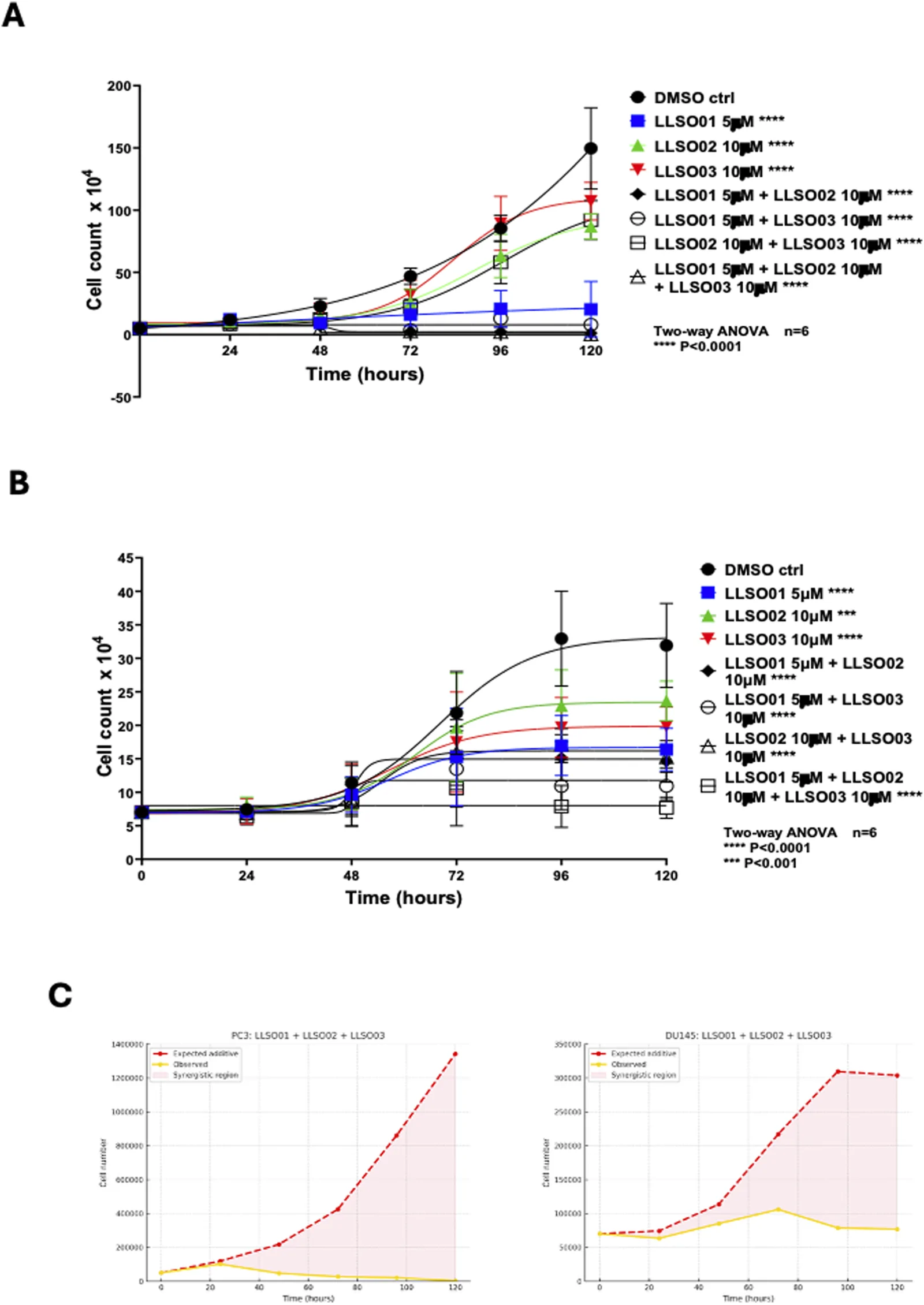
The cumulative effect of the LLSOs on the growth of PC3 and DU145. **(A)** Growth curve of PC3 cells treated with a combination of LLSO or DMSO. All LLSOs treatment either on their own or in different combinations inhibited the growth of PC3 cells. Treated PC3 cells with DMSO control, 5 µM LLSO1, 10 µM LLSO2, 10 µM LLSO3, 5 µM LLSO1 + 10 µM LLSO2, 5 µM LLSO1 + 10 µM LLSO3, 10 µM LLSO2 + 10 µM LLSO3 or 5 µM LLSO1 + 10 µM LLSO2 + 10 µM LLSO3 after cells were seeded in 24-well plates for 24 h. Medium with LLSOs treatment was refreshed every 48 h, and cells were counted every 24 h after 24 h of seeding PC3 cells for a total of 120 h. Two individual experiments with three repeats for each treatment in each individual experiment, data was analysed by two-way ANOVA. n = 6, ****p < 0.0001. **(B)** Growth curve of DU145 cells treated with a combination of LLSO or DMSO. LLSOs had a cumulative effect on DU145 cell growth, showing greater inhibition of cell growth in any combination compared with the treatment of any of the three LLSOs alone. Treated DU145 cells with DMSO control, 5 µM LLSO1, 10 µM LLSO2, 10 µM LLSO3, 5 µM LLSO1 + 10 µM LLSO2, 5 µM LLSO1 + 10 µM LLSO3, 10 µM LLSO2 + 10 µM LLSO3 or 5 µM LLSO1 + 10 µM LLSO2 + 10 µM LLSO3 after cells were seeded in 24-well plates for 24 h. Medium with LLSOs treatment was refreshed every 48 h, and cells were counted every 24 h after 24 h of seeding DU145 cells for a total of 120 h. Two individual experiments with three repeats for each treatment in each individual experiment, the data was analysed by two-way ANOVA. n = 6, ***p < 0.001, ****p < 0.0001. **(C)** Bliss independence analysis in both PC3 and DU145 cells showed synergy of the 3 compounds.

To determine whether LLSOs have cumulative/synergistic effects, the growth curves in PC3 and DU145 were repeated by treating the cells with combinations of LLSOs. As seen in Figure 4A, all different combinations of LLSOs significantly reduced the growth of PC3 cells. Treatment with LLSO1 alone almost completely abolished growth of PC3 cells, making it difficult to determine whether combined LLSO1 + LLSO2/3 treatment had an additive effect. We also found combined treatment with LLSO2 and LLSO3 increased growth inhibition compared to LLSO3 alone. However, this growth inhibition was comparable to LLSO2 alone. Therefore, in PC3 cells, the cumulative effect of LLSOs on growth was unclear.

We also evaluated these drugs in DU145 cells (Figure 4B). We found treatment of DU145 cells with all three LLSO compounds simultaneously resulted in the strongest growth inhibition, completely abrogating cell proliferation. Bliss independence analysis revealed synergistic interactions among the three compounds (Figure 4C), as well as between various pairs of LLSOs in both PC3 and DU145 cells (Supplementary Figure S12).

### 3.4 The effects of LLSO3 on tumour growth in prostate cancer xenograft models in nude mice

**FIGURE 5:**
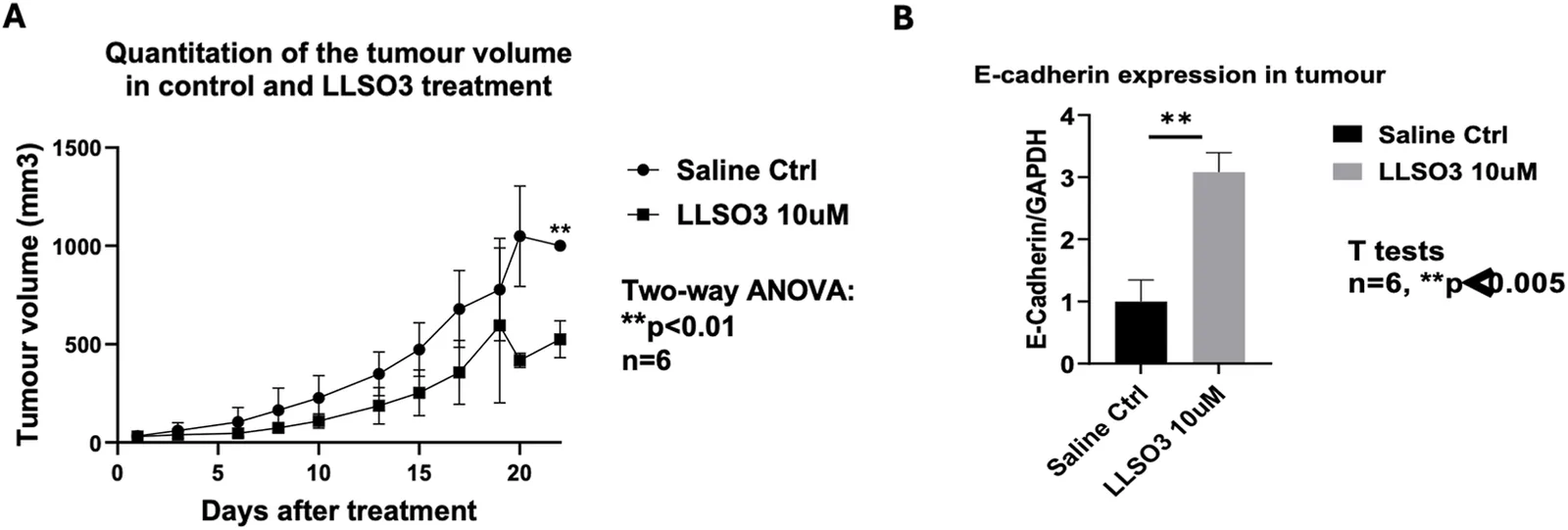
10μM LLSO3 inhibit tumour growth in PC3 xenografts in nude mice by increasing E-cadherin levels. **(A)** Quantitation of the tumour volumes in saline (control) and 10 mg/kg LLSO3 treated mice at different times post-treatment. Tumour volumes were estimated using the formula ‘volume = [(length + width)/2] x length x width’. Quantitation of the tumour volumes was analysed by Two-way ANOVA using GraphPad Prism9. **(B)** qRT-PCR for E-cadherin expression was performed with extracts from control and 10 mg/kg LLSO3 treated PC3 tumours. Normalization of the relative gene expression using GAPDH as the reference control. There are two repetitions within each of the 3 independent tumour samples. n = 6. Results were expressed as Mean ± SD, *p < 0.05, **p < 0.01.

LLSO3 was selected for *in vivo* evaluation because it showed consistent EMT-associated effects *in vitro*, reduced prostate cancer cell migration, was less strongly cytotoxic than LLSO1 under the tested conditions, and corresponds to a clinically approved opioid receptor antagonist with an established safety profile. LLSO2 has previously been reported to exert anti-proliferative effects in cancer models (Li et al., 2022), and therefore represented a less novel candidate for *in vivo* validation in the present study. In contrast, LLSO3 demonstrated the most consistent EMT-reversal phenotype *in vitro*, including robust upregulation of E-cadherin expression and suppression of migratory capacity across prostate cancer cell lines. Notably, LLSO3 corresponds to Naltrexone hydrochloride, a clinically approved μ-opioid receptor antagonist with an established safety profile in humans, enhancing its translational potential. Based on its strong EMT-modulating activity and favourable tolerability profile, LLSO3 was selected for *in vivo* evaluation. Tumour growth was therefore assessed in PC3 xenografts established in nude mice. Supplementary Figures S13A,B show the tumour growth traces in individual mice. As shown in Figure 5A, 10 mg/kg LLSO3 inhibited tumour growth compared with saline-treated controls in PC3 xenografts in nude mice.

In order to clarify whether LLSO3 inhibits PCa tumour growth through regulation of EMT *in vivo*, the expression level of E-cadherin in tumour xenografts was investigated by qPCR using RNA extracted from excised tumour tissues. These data from Figure 5B demonstrate that LLSO3 can reduce PC3 xenograft tumour growth and is associated with increased expression of an epithelial marker E-cadherin in tumour tissue.

### 3.5 The effects of LLSOs compounds on EMT properties in different cancer types

**FIGURE 6:**
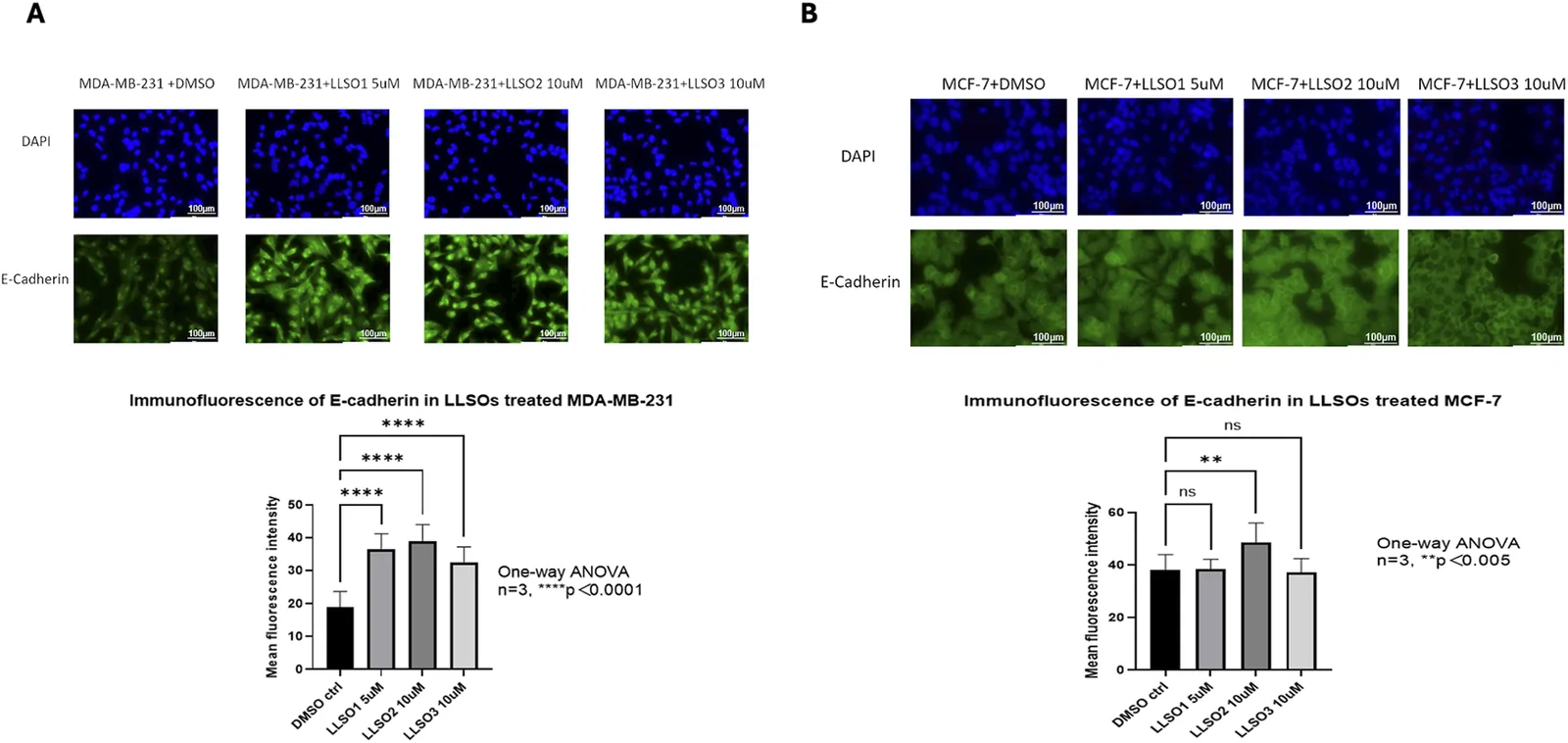
All LLSOs increased the expression of E-cadherin in MDA-MB-231 cells while only LLSO2 increased it in MCF-7. **(A)** Immunofluorescence analysis for E-cadherin expression was performed following 48 h treatment of MDA-MB-231 cells with DMSO, 5 μM LLSO1, 10 μM LLSO2, and 10 μM LLSO3. MDA-MB-231 cells stained with mouse IgG were used as negative control and LNCaP cells were used as a positive control. All three LLSOs showed an increase in the E-cadherin expressing level. There are three wells repeated within each of the two independent experiments, n = 3. Quantification of the E-cadherin signal was performed with ImageJ by using 9 fields of 3 repeat slides for each treatment. Photomicrographs were taken at ×200 magnification; Scale bar is 100 µm. **(B)** Immunofluorescence analysis for E-cadherin expression was performed following 48 h treatment of MCF-7 cells with DMSO, 5 μM LLSO1, 10 μM LLSO2, and 10 μM LLSO3. MCF-7 cells stained with mouse IgG were used as negative control and LNCaP cells were used as a positive control. LLSO2 induced an increase in the expression level of E-cadherin, whereas LLSO1 and LLSO3 showed no effect on E-cadherin expressing level. There are three wells repeated within each of the two independent experiments, n = 2. Quantification of the E-cadherin signal was performed with the ImageJ software by using 9 fields of 3 repeat slides for each treatment. Photomicrographs were taken at ×200 magnification; Scale bar is 100 µm.

Although the primary focus of this article is prostate cancer, we also explored whether LLSO-associated induction of E-cadherin occurred in selected non-prostate cancer cell lines. MCF-7 and MDA-MB-231 breast cancer cells, CALU-3 lung cancer cells, HCT-116 colorectal cancer cells and SKOV-3 and A2780 ovarian cancer cells were treated with LLSO compounds and analysed by immunofluorescence. Induction of E-cadherin by all three LLSOs was observed only in the MDA-MB-231 breast cancer cell line (Figures 6A,B), whereas in MCF-7 cells, only LLSO2 induced E-cadherin expression. No induction was detected in lung, colon, or ovarian cancer cell lines (CALU-3, HCT-116, SKOV-3, A2780; Supplementary Figures S14 and S15).

These results suggest that LLSOs-associated modulation of E-cadherin is context-dependent and not universal across all cancer cell lines.

### 3.6 Mechanism through which LLSOs compounds induce MET in PCa cells

In a previous paper, we investigated the mechanism by which LLSO2 induces MET (Li et al., 2022). Here, we further characterized the pathways for LLSO1 and LLSO3 that may contribute to EMT-associated effects through inhibitor-based experiments in PC3 cells and assessed E-cadherin expression by immunofluorescence.

#### 3.6.1 LLSO1 signalling

As illustrated in the hypothetical LLSO1 signalling pathway depicted in Supplementary Figure S16, TTCC (T-type Calcium Channel) triggers signalling pathways through the elevation of intracellular Ca^2+^. After an influx of Ca^2+^, the calmodulin (CaM) and the downstream effector calmodulin kinase II (CaMKII), which are the proven transducers of TTCC activity, are activated (Welsby et al., 2003). Furthermore, studies have revealed that the Ras/Raf/MEK/ERK pathway is transiently activated after the activation of TTCC through the CaMKII, whereas inhibition of TTCC blocks the Akt/PKB pathway. Both signalling pathways were involved in the progression of DNA synthesis included in the cell cycle through controlling the cyclin/cyclin-dependent kinase (CDK) complexes (Choi et al., 2005; Zhang et al., 2017; Sallán et al., 2018). Moreover, Wang et al. also found that c-Src non-receptor tyrosine kinases function downstream of CaMKII in a signaling cascade and plays a critical role in many aspects of cancer progression including proliferation, migration and invasion in various cancer types (Zhang and Yu, 2012; Wang et al., 2003). The CaMKII and CaMKIV activated by Ca2+/CaM were also found to be able to activate the cyclic AMP response element binding protein (CREB), overexpressed in diverse solid tumour types such as NSCLC, BrCc and glioblastoma, and have oncogenic potential by regulating a variety of cellular responses, including growth, proliferation, and survival (Steven and Seliger, 2016). Besides CREB, the transcription factor nuclear factor of activated T-cell (NFAT) could be activated by promoting activation of the well-known calcium and calmodulin dependent protein serine/threonine phosphatase calcineurin (CaN) through the influx of Ca^2+^ (Rusnak and Mertz, 2000; Macian, 2005).

The mechanism by which LLSO1 signals to increase the expression level of E-cadherin was determined by using the immunofluorescence described above. CaM as a calcium-sensing protein, has more than 200 distinct substrates, and the essential targets of CaM-dependent pathways required for cell proliferation remain largely elusive (Klee et al., 1980; Yap et al., 2000). Hence, one of the key regulators of intracellular calcium signalling, CaN, was first targeted as activation of CaN has been considered to play a pro-tumorigenic role, which is linked to increased cell activity, including promoting proliferation, migration, and invasion in diverse cancers like BrCc, PCa, LUAD and COAD (Brun and Godbout, 2016).

**FIGURE 7:**
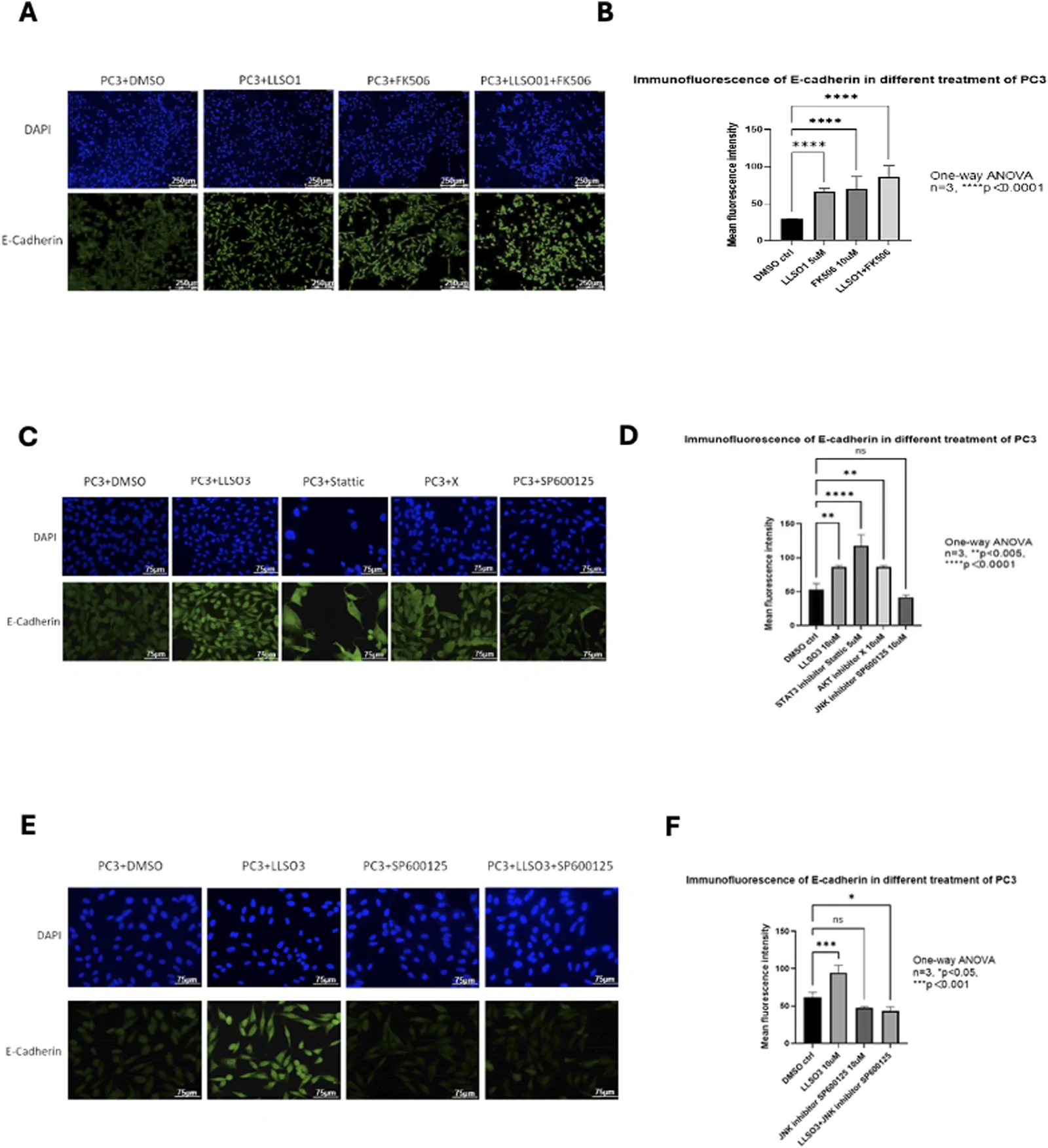
Signalling pathways used by LLSO1 and LLSO3 in PC-3 cells. *Left panel*: Inhibition of CaN increased E-cadherin expression in PC3 cells. **(A)** Images of PC3 cells treated separately with 10 μM of LLSO1, 40 μM of FK506, and the combination of LLSO1 and FK506. All images were taken at ×100 magnification. The scale bar is 250 µm. Representative examples from at least 9 microscopic fields per experiment, experiments repeated 3 times with triplicate per treatment. **(B)** Quantification of immunofluorescence for E-cadherin expression was performed after 48 h of treatments of PC3 cells with DMSO, 10 μM of LLSO1, 20 μM of FK506, or the combination of LLSO1 and FK506. All these treatments showed an increase in the level of E-cadherin expression. n = 3, data was analysed by one-way ANOVA. *Middle panel*: Inhibition of STAT3 increased E-cadherin expression in PC3 cells, but not inhibition of AKT or JNK. **(C)** Images of PC3 cells treated separately with 10 μM of LLSO3, 5 μM of STAT3 inhibitor Stattic, 10 μM of AKT inhibitor X, and 10 μM of JNK inhibitor SP600125. All images were taken at ×200 magnification. The scale bar is 250 µm. Representative examples from at least 9 microscopic fields per experiment, experiments repeated 3 times with triplicate per treatment. **(D)** Quantification of immunofluorescence for E-cadherin expression was performed after 48 h treatments of PC3 cells with DMSO, 10 μM of LLSO3, 5 μM of STAT3 inhibitor Stattic, 10 μM of AKT inhibitor X, and 10 μM of JNK inhibitor SP600125. All these treatments showed an increase on E-cadherin expression level. n = 3, data was analysed by one-way ANOVA. *Right panel*: LLSO3 uses JNK signalling pathway to increase E-cadherin expression in PC3 cells. **(E)** Images of PC3 cells treated separately with 10 μM of LLSO3, 10 μM of AKT inhibitor X, the combination of LLSO3 and X, 10 μM of JNK inhibitor SP600125, and the combination of LLSO3 and SP600125. All images are taken at ×200 magnification. The scale bar is 250 µm. Representative examples from at least 9 microscopic fields per experiment, experiments repeated 3 times with triplicate per treatment. **(F)** Quantification of immunofluorescence for E-cadherin expression was performed after 48 h treatments of PC3 cells with DMSO, 10 μM of LLSO3, 10 μM of AKT inhibitor X, the combination of LLSO3 and X, 10 μM of JNK inhibitor SP600125, and the combination of LLSO3 and SP600125. All these treatments showed an increase on E-cadherin expression levels except for the treatment of 10 μM of JNK inhibitor SP600125, and the combination of LLSO3 and SP600125. n = 3, data was analysed by one-way ANOVA.

The widely used CaN inhibitor, FK506 (Tacrolimus), has been shown to suppress the proliferation and migration of several cancers including PCa and BrCc (Akool el et al., 2008; Periyasamy et al., 2007; Goshima et al., 2019). We therefore treated PC3 cells with FK506, alone and in combination with LLSO1, to assess whether inhibition of CaN recapitulates the effects of LLSO1 on E-cadherin expression. As shown in Figures 7A,B, FK506 treatment—whether alone or with LLSO1—led to increased E-cadherin expression, comparable to that observed with LLSO1 alone. These findings suggest that LLSO1 may exert its effects through the CaN signaling pathway.

#### 3.6.2 LLSO3 signalling

An increasing number of studies have described that several opioid receptors and receptor subtypes are widely implicated in various types of cancer cells and are associated with cancer aggressiveness, particularly G-protein-coupled receptors, including mu-opioid receptor (MOR), kappa-opioid receptor (KOR), and delta-opioid receptor (DOR) (Scroope et al., 2021; Carli et al., 2020). Based on the illustrated hypothetical signalling pathway depicted in Supplementary Figure S17, it is possible that LLSO3, functioning as a non-selective opioid receptor antagonist, possesses the potential to affect all these pathways. Since STAT3 and AKT both have the ability to regulate certain types of cancer progression through modulating EMT, and are both present in all three opioid receptor pathways, whether STAT3 and AKT are involved in the mechanisms of LLSO3 inhibiting PCa cell growth and migration, and EMT regulation were firstly assessed (Wendt et al., 2014; Xu et al., 2015). In addition, a growing body of studies have indicated that activation of JNK plays a critical role in inducing EMT and promoting migration and invasion in several cancer cell types, including PCa, BrCc, and COAD (Arisan et al., 2020; Wang et al., 2010; Meng et al., 2019). Therefore, whether STAT3, AKT and JNK are involved in the effect of LLSO3 on EMT regulation were also investigated by using Stattic as a STAT3 inhibitor, X as an AKT inhibitor, and SP600125 as a JNK inhibitor.

As shown in Figures 7C,D, treatment of PC3 cells with both Stattic and LLSO3 increased E-cadherin expression above the level of LLSO3 alone, indicating that STAT3 inhibition may play a role in mediating LLSO3’s effect. In contrast, treatment of PC3 cells with both LLSO3 and AKT had no effect on E-cadherin expression, whilst co-treatment with LLSO3 and JNK prevented the LLSO3-induced upregulation of E-cadherin (Figures 7E,F). These findings suggest that LLSO3 likely exerts its EMT-inhibitory effects via the JNK signalling pathway rather than the AKT pathway.

## 4 Discussion

EMT plays a significant role in cancer progression that includes metastasis and invasion. EMT is also critical in drug resistance acquisition in cancer treatment and in immunosuppression and refractory responses to chemotherapy. Consequently, EMT has become a highly valued target for the development of novel cancer therapeutic strategies. EMT-targeting therapeutic strategies may be able to prevent cancer cell invasion and dissemination, as well as reduce cancer stemness and increase the efficacy of traditional chemotherapeutics (Jonckheere et al., 2022).

Several EMT-targeting strategies have been explored in recent years, including blocking key signalling pathways (e.g., TGF-β, Wnt, Notch, Hedgehog, MAPK/ERK, Hippo, and hypoxia signalling pathway) (Gonzalez and Medici, 2014; Shetty et al., 2020; Mendonsa et al., 2018; Loh et al., 2019; Zang et al., 2017); modulating EMT-associated MicroRNAs (miRs) (La et al., 2013; Zaravinos, 2015; Park et al., 2008; Korpal et al., 2008); and inhibiting the EMT-inducing transcription factors (EMT-TFs) such as Snail1/2, ZEB1/2, Twist1/2 and LEF-1 (Gonzalez and Medici, 2014); as well as regulating EMT markers, like E-cadherin, cytokeratin, N-cadherin, β-catenin, vimentin, and fibronectin (Hirano et al., 2013; Tanaka et al., 2010; Thaiparambil et al., 2011; Wang et al., 2016; Ramamurthy et al., 2018). However, clinical translation of these approaches has been limited. EMT transcription factors are largely considered “undruggable” due to their nuclear localization and lack of enzymatic domains, while miRNA-based therapies face delivery and stability challenges, as well as off-target effects (Bushweller, 2019; Testa et al., 2019; Lambert et al., 2018; Rupaimoole and Slack, 2017). Furthermore, inhibition of central pathways such as TGF-β has been associated with systemic toxicity owing to their critical roles in normal tissue homeostasis, wound repair, and immune regulation (Massague and Sheppard, 2023; Tauriello et al., 2022).

In contrast, the LLSO compounds investigated in this study appear to regulate EMT through modulation of upstream signalling environments rather than directly inhibiting core EMT transcription factors. LLSO1 (NNC-55-0396 dihydrochloride) and LLSO2 (Nemadipine A) target voltage-gated calcium channels, implicating calcium-dependent signalling cascades in EMT plasticity. Calcium signalling is known to influence transcriptional programs, cytoskeletal dynamics, and cell migration, all of which are central to EMT progression (Adiga et al., 2022; Monteith et al., 2017). Meanwhile, LLSO3 (Naltrexone hydrochloride) acts through μ-opioid receptor blockade and was associated in our study with modulation of JNK/STAT3 signalling pathways previously implicated in EMT maintenance and cancer cell motility. Thus, rather than directly suppressing EMT-TFs or altering miRNA expression, LLSO compounds may attenuate EMT by interfering with signalling networks that sustain mesenchymal phenotypes.

This signalling-level intervention may represent a complementary and potentially more druggable strategy compared with direct targeting of nuclear EMT drivers. Given the absence of clinically validated EMT-targeted therapies in prostate cancer, modulation of calcium and opioid receptor–associated pathways may offer an alternative approach for limiting EMT-associated tumour progression.

The observed increase in E-cadherin and reduction in N-cadherin/Twist are consistent with a shift toward a less mesenchymal phenotype. However, EMT is a complex, dynamic and partial process, and no single marker set is sufficient to prove complete EMT reversal. The current data support modulation of EMT-associated phenotypes rather than definitive induction of a full mesenchymal-to-epithelial transition. Future studies should assess additional epithelial markers, such as cytokeratin 8 and cytokeratin 18; mesenchymal markers, such as vimentin and fibronectin; extracellular matrix-associated markers, such as laminin; and invasion through matrix-containing barriers.

The migration data provide the strongest functional support for an EMT-associated phenotype. All three LLSO compounds reduced migration of LNCaP and DU145 prostate cancer cells. This finding is important because EMT is functionally linked to motility and metastatic potential. Nevertheless, reduced migration can arise from several mechanisms, including altered adhesion, impaired cytoskeletal dynamics, reduced viability, slower proliferation or specific EMT modulation. The viability and apoptosis assays suggest that cytotoxicity alone does not fully account for the migration findings, particularly because acute viability was not consistently reduced across all treated PCa cell lines.

The *in vivo* data identify LLSO3as the most translationally interesting compound in this study. LLSO3 treatment at 10 mg/kg reduced PC3 xenograft tumour growth and was associated with increased E-cadherin expression in excised tumours. The selection of LLSO3 for the xenograft study was based on its consistent *in vitro* EMT-associated effects, reduced migration, lower overt cytotoxicity compared with LLSO1 under the tested conditions, and its status as a clinically used opioid receptor antagonist. These features provide a rationale for drug repurposing. However, additional *in vivo* studies are required before therapeutic conclusions can be drawn. These should include histological assessment of tumour morphology, immunohistochemistry for EMT markers, angiogenesis markers and proliferation/apoptosis markers, direct assessment of opioid receptor pathway activity, and evaluation in orthotopic or metastatic PCa models.

The inhibitor-based experiments provide preliminary mechanistic clues. For LLSO1, the effect of FK506 is compatible with involvement of calcineurin-associated signalling, but direct evidence of T-type calcium channel inhibition, altered intracellular calcium flux, calcineurin activity or NFAT localisation was not provided. For LLSO3, STAT3, and JNK-associated observations suggest possible pathway involvement, but the experiments were performed only in PC3 cells and relied on pharmacological inhibitors. Direct pathway validation will require Western blotting or phospho-flow analysis to assess pathway activation, genetic knockdown or rescue of relevant targets, and validation in additional prostate cancer cell lines, such as DU145 and LNCaP.

The role of opioid receptor signalling in cancer remains context-dependent. Published studies have linked Mu-opioid receptor signalling to proliferation, migration and EMT-associated phenotypes in lung cancer and other models (Lennon et al., 2014), while delta-opioid receptor signalling has been implicated in breast cancer metastasis through JAK1/2-STAT3 signalling (Tripolt et al., 2021). Low-dose naltrexone has also been reported to inhibit EMT in cervical cancer model (Liu et al., 2021). Conversely, other work suggests that repeated naltrexone treatment can promote EMT-associated behaviour in bladder cancer cells (Wang et al., 2021). These apparently divergent findings reinforce the need to define dose, exposure schedule, receptor expression pattern and tumour context when interpreting the effects of naltrexone on EMT-associated phenotypes. In PC3 xenografts, our data support an inhibitory effect on tumour growth and increased E-cadherin expression, but the precise receptor-dependent mechanism remains to be established.

Compared with direct EMT transcription factor targeting, the LLSO approach may be more pharmacologically tractable because it acts through druggable upstream signalling nodes. This study identifies LLSO1 (NNC-55–0396 dihydrochloride), LLSO2 (Nemadipine A) and LLSO3 (Naltrexone hydrochloride) as pharmacologically distinct modulators of EMT-associated phenotypes in prostate cancer cells. The compounds increased epithelial marker expression, reduced mesenchymal-associated marker expression in selected assays, and suppressed prostate cancer cell migration. LLSO3 further reduced PC3 xenograft tumour growth and increased E-cadherin expression in tumour tissue *in vivo*.

These findings support further investigation of calcium channel-associated and opioid receptor-associated signalling as potentially actionable regulators of EMT-associated prostate cancer progression. LLSO3/Naltrexone hydrochloride is a particularly attractive candidate for future study because of its clinical availability. However, further studies are required to fully elucidate the molecular mechanisms underlying LLSO-mediated EMT regulation. Future work should also explore combination strategies with existing prostate cancer therapies, particularly given the observed synergistic effects *in vitro*.

## Data Availability

The original contributions presented in the study are included in the article/Supplementary Material, further inquiries can be directed to the corresponding author.
